# Professor Mattrucci on the Physical Phenomena of Living Beings

**Published:** 1847-04

**Authors:** 


### ART. IV.

*Leçons sur les Phénomènes Physique des Corps Vivants.* Par C. MATTEUCCI. Edition Française, publiée, avec Additions considérables, sur la Deuxième Edition Italienne.—*Paris,* 1847. Avec 18 figures.

*Lectures on the Physical Phenomena of Living Beings.* By C. MATTEUCCI. French Edition, translated, with considerable Additions, from the Second Italian Edition.—*Paris,* 1847. 12mo, pp. 406.

IN the year 1844, the Government of Tuscany charged Professor Matteucci with the duty of delivering at the University of Pisa a series of lectures on the Physical Phenomena of Living Beings. In establishing a distinct course upon this subject, the Tuscan Government exhibited a degree of enlightenment, which we should be glad to see reflected by the bodies that have the control of medical education in this country. No less sagacious was it in the choice of a professor; for it would have been difficult if not impossible, to find anywhere a man better fitted for such an office than M. Matteucci, who unites the highest experimental skill with extensive knowledge of this class of subjects, and who combines, in a very rare degree, the acuteness of a practised observer with the sagacity and generalizing powers of a true philosopher. His lectures were immediately published in Italy; and in a second edition, which was speedily called for, very extensive additions were introduced. The French translation has been made from the second edition, under the supervision of the author, who, from his long residence in Paris (where he acquired much of his proficiency in physical science), may be considered almost half a Frenchman; and he has made it the vehicle for the publication of his latest views on many of the subjects to which he has of late more particularly devoted his attention, including those which he brought forward at the late meeting of the British Association at Southampton. Consequently this French edition may be regarded as possessing the authority of an original work, instead of being crowded with the errors which too often disfigure such reproductions, especially among our Gallic neighbours.

We attach peculiar importance to the appearance of this little volume, for reasons which will more fully appear as we proceed. The lectures are in subject and style just what might be advantageously addressed to a class of students; whilst their conciseness will prevent them from taxing his powers of attention to any considerable extent. The only fault that we have to find with them, is that they go into somewhat too much of detail on the author's favorite subject, Electricity; and that other topics are consequently handled with undue brevity. We cannot but hope that they may be given to the British public in some shape or other; and this defect might then be easily rectified.

On the importance of a due acquaintance with the physical phenomena of living beings, as well to the scientific physiologist and pathologist, as to the practitioner who aims at nothing higher than the skilful application of the therapeutic art, a very few prefatory words will suffice. It has been elsewhere pointed out that there are in the living body three classes of phenomena; the first of them obviously *physical,* and not in any way modified or checked by the vital operations, such as the action of muscles upon bones according to the laws of leverage, or the propulsion of the blood through the large vessels by the force-pump action of the heart equalized by the elasticity of their walls; the second class as obviously *vital*, being altogether different from anything which is seen in physics or chemistry, and referrible to a distinct category, such as the whole process of cell-development and reproduction, which may be considered as the fundamental type of this class of operations; and thirdly, an intermediate group, in which physical laws appear to be operating under peculiar conditions, which scarcely anything except a living organized body can supply, such as the change of venous into arterial blood in the pulmonary capillaries, the production of animal heat, and many chemical transformations. Of this last class of phenomena, a large proportion have been ranked in the second division; and by some physiologists they are still referred to it, the results appearing to them too far removed from those of any ordinary physical or chemical principles to be reasonably attributed to them. But although there are many phenomena, in regard to whose character doubts must long remain,—the peculiarity of the conditions under which they occur, making it difficult to imitate them experimentally,—there can be no question that the present tendency of chemical and physical inquiry is to limit more and more the domain of the peculiarly vital operations, by showing that wherever living beings come into contact (so to speak) with the external world, the laws of action which are dominant in the latter, exert their full influence in the bodies of the former; and that it is only within the *penetralia* of the system that the purely vital laws have free scope. Thus, to commence with the act of digestion, no intelligent physiologist any longer entertains a doubt that it is to be entirely explained on chemical principles; a conclusion which might have been anticipated from the simple anatomical fact, that the food whilst still within the intestinal canal is really on the exterior of the body, and can scarcely be regarded as more under the influence of vital operations than if it were in contact with the skin. Whilst a part of the alimentary materials undergoes simple solution, another portion undergoes very important transformations; and the nature of these is gradually being elucidated by experimental researches, whose whole plan is to imitate as much as possible the conditions under which the phenomena take place in the living body, and whose whole success depends on the degree in which that imitation is effected. Of this we shall presently meet with striking examples, when we come to notice the results of late inquiries into the mode in which fatty matters are reduced to a state fit for absorption, and in which amylaceous substances are transformed into sugar, lactic acid, or even into fat. In regard, again, to the absorption of these products, or their introduction into the living system, everything tends to show that a large part of the process takes place in strict conformity with physical principles; that which appears, from the observations of Mr. Goodsir, to be due to the vital operation of cell-growth, being probably far inferior in amount to that which takes place by simple imbibition, which, as the researches of Professor Matteucci have shown, will legitimately account for many phenomena previously supposed to be beyond the scope of its operations. When we examine these researches, we shall find that their success is due to the same method of conducting them as that which has been so successful in Organic Chemistry; namely, the imitation, so far as is practicable, of the conditions under which the phenomena occur in the living body.

As soon, however, as the alimentary matter has been introduced into the circulating system it is subjected to a new set of influences. There is no check to the purely chemical transformations which were previously going on; but a new power comes into play, by which, with little or no appreciable change in the chemical constitution of certain of the nutritive materials, they are caused by a new arrangement of their components to present new properties, the crude unorganizable albumen being converted into spontaneously fibrillating plastic fibrine. Now we would not venture to assert dogmatically that this is a change never to be accounted for by any other than vital agency; but such is the view we are at present inclined to take of it,—the change being one which is peculiar to the living body, alike in its character, in its purposes, and in the mode in which it is effected. In its character, since we meet with no substance in the inorganic world, which is at all analogous to fibrine in its properties; in its purposes, since this transformation is evidently but a stage of preparation for those processes of more perfect organization which are altogether peculiar to living bodies; and in the mode in which it is effected, because there appears to be sufficient evidence for referring the transformation to the growth, development, and disintegration of successive generations of cells floating in the circulating fluid, in which, as we shall next attempt to show, the very essence of vital operations consists. The circulating fluid, having undergone its requisite preliminary elaboration, is converted by the process of nutrition into living organized tissues, endowed with various properties; some of which are simply physical (such as elasticity, penetrability by liquids, and the like); whilst others are unlike those which we meet with in any form of inorganic matter, and are, therefore, termed vital, for the sake of distinction (such as contractility, sensibility, &c.) Now, as we have argued on several former occasions, all these properties are alike in this,—that they are entirely dependent upon the peculiar arrangement of the material substances by which they are exhibited, and disappear as soon as that arrangement has ceased to exist; but they also differ in this,—that the physical properties may exist in forms of matter purely inorganic (as elasticity in steel, penetrability to liquids in sandstone), whilst the vital properties can only manifest themselves in structures that have been generated by a living body, and are maintained under conditions which it alone can supply (the contractility of muscle continuing to manifest itself only for a short time after the cessation of the circulation of arterial blood through it, and the sensibility of the nervous tissue being immediately suspended by any check to that function).

As the cell is the simplest type of an organized structure, and as the whole process of nutrition in the most complex organisms appears referrible to the laws of cell-development, we need not go beyond this, in examining how far it is possible to account for that process on purely chemical and physical principles. Although we here differ from Professor Matteucci, who seems to think that our knowledge of these will go far to explain the phenomena of cell-growth, we are bound to express our decided conviction that the most important and characteristic of these phenomena belong to a category altogether distinct. There is, it is true, a certain analogy between the transforming power that a cell-germ appears to possess over the elements upon which it operates, (especially in the vegetable kingdom), and the *catalytic* action of many substances with which the chemist is familiar; there is a certain conformity between the phenomena of endosmose, and the gradual enlargement of a cell by the penetration of liquid into its interior; and there is a decided resemblance between the apparently homogeneous membrane which forms the cell-wall, and the pellicle which has been shown by Ascherson to be produced whenever oily matter and albumen come into contact. But even if it were granted (which we are not yet ready to do) that the selection of the peculiar contents of the cell and the transformation effected in them is a simple result of the chemical constitution of the germ or nucleus,—that the enlargement of the molecular point, in which no cavity can be detected, to a perfect cell, is due to the penetration of the matters thus attracted, according to the physical laws of imbibition—and that the cell-wall is merely the product of the contact of oil and albumen—we still ask what there is in the inorganic world which bears the remotest resemblance to the phenomenon of *reproduction* even in its simplest form (that in which the offspring constantly resembles its parent), still less in that extraordinary manifestation of it which we see in the course of the embryonic development of the higher animals, where the primitive cell first produces a congeries apparently similar to itself, but where this homogeneous mass gradually developes itself into an assemblage of heterogeneous organs and tissues, differing alike from each other and from their common ancestor in form and composition, but all having the most intimate relations of mutual dependence, and forming one perfect organism of high complexity, resembling that by which the germ was originally produced. All this appears to us so utterly devoid of the remotest analogy in the inorganic world, as to have an unquestionable claim to rank as a distinct order of phenomena; and we cannot stretch our imagination so far as even to glimpse at the possibility of including them legitimately in the same generalization with those of physics and chemistry.

But when we pass from the *constructive* to the *destructive* operations which are continually going on in the living body, we return to the domination of physics and chemistry. As soon as the process of disintegration commences in any tissue, the products of that disintegration seem to be no others than those which would elsewhere take place with the same materials placed under the same circumstances. Thus carbonic acid, which is the one most constantly generated of all these products in the living body, is the one which is most invariably produced by the decomposition of organized matter removed from it; and although the conditions of the production of urea, lithic acid, fæcal matter, and other excrementtitial substances, are not yet fully understood, yet the demonstration which has been given of the possibility of generating them artificially, shows that the affinities which cause their elements to combine in the proportions and modes severally peculiar to them can be no other than chemical. Of the act of respiration, by which the carbonic acid of the system is set free, and that measure of oxygen is introduced which is requisite to excite the various operations of the tissues by its chemical action upon them, it is almost superfluous to remark that it is a process of a purely physical character; its peculiarity chiefly consisting in the state of extremely minute division in which the blood is exposed to the air, and in the peculiar power of absorbing gases which that liquid possesses. Of the process of secretion, which brings us back again from the penetralia of the organism to the external world, we do not yet know enough to separate clearly the phenomena which are due to simple transudation from those which are the result of the vital act of cell-growth; and here there is a wide field open for inquiry which is almost sure to be productive. Many of the fluids poured forth within the living body agree so closely with the serous portion of the blood in their general characters, that it would appear scarcely necessary to attribute their separation to anything else than an act of transudation; whilst for those which are more particularly destined to be cast at once out of the body, and which consist chiefly of substances alien to the normal constitution of the blood, the selecting power of cells appears to be required.

From these preliminary remarks, we trust it will appear that no scientific physiologist can have any excuse for neglecting to make himself acquainted with all that physics can contribute to the elucidation of the phenomena of living beings; and that such knowledge is also of the highest value to the practitioner, whose influence is chiefly, if not solely, exerted over those processes in which physics and chemistry have the greatest share. He cannot himself reach the mysterious *penetralia* of the system; but he can tread its outer courts, and exercise much control over the ingress, and watch the egress of its ministers; and in proportion as he becomes acquainted with the respective offices of these, may he regulate at his will all that takes place within.

We shall now enter upon a somewhat detailed examination of Professor Matteucci's treatise; confining ourselves, however, almost entirely to those topics which are least perfectly treated in physiological works, and especially dwelling on his original observations. After a general introduction, the first subjects discussed by Professor Matteucci are Capillary Attraction, Imbibition, Endosmose, and Absorption; which are all referrible to the same category, and a due acquaintance with the physical phenomena of which is indispensable to the physiologist who would seek to determine the purely vital powers concerned in the introduction of fluids into the living tissues. As these topics are handled with peculiar skill, and many new and important observations are brought forwards, in the treatise before us, we shall dwell upon them somewhat at length.

We should scarcely think it necessary to remind our readers of the ordinary phenomena of Capillary Attraction, to which Professor Matteucci adverts in the first instance, did we not know that many vague and incorrect notions are prevalent respecting it. When a solid body is plunged into a liquid, the latter is *elevated* or *depressed* around the solid, according as the substance of the latter *is*, or *is not*, moistened by the liquid; thus, if a glass rod be dipped into water, the surface of liquid will be elevated around it, whilst, if it be dipped into mercury, the surface will be depressed. In the same manner, if a tube of small diameter and open at both ends be plunged into the liquid, this will be raised or depressed in a degree which increases with the diminution of the diameter of the tube; thus, in a tube of 1 millimeter in diameter there will be an elevation of water to the height of 30 millimeters, whilst there will be a depression of mercury to the depth of 13 millimeters. That this force should play an important part in the introduction of liquids into living tissues will be easily perceived, when it is borne in mind that the parietes of their vessels are peculiarly susceptible of being moistened by aqueous fluids, and that their calibre is commonly not more than from 1-100th to 1-200th of a millimeter. (A millimeter is about 1-25th of an inch.) These phenomena are entirely independent of the thickness of the walls of the tubes; they are not influenced by the pressure of the surrounding air, but will take place equally in a rarefied or condensed atmosphere, in any other gaseous medium, or in a perfect vacuum. They are influenced, however, by temperature; the elevation or the depression of the column of liquid diminishing with an increase of heat, the tube and liquid remaining the same. The degree of elevation or depression has no relation with the density of the liquid; thus, if we take water as the standard, and represent its elevation in a given tube by 100, that of alcohol will be 40, and of essence of lavender 37, whilst that of a saturated solution of common salt will be 88. It is very important to bear in mind that simple capillary attraction will never cause a liquid to *flow over* from the top of a tube, although its summit be much lower than the elevation which the same liquid would attain in a tube of corresponding diameter; this is easily understood when it is borne in mind that there is nothing to continue attracting the fluid, when once it has been raised to the summit of the tube. The case is very different, however, if the liquid be carried off by any agency existing at the end of the tube; for it will then be drawn up and replaced as fast as it is removed. This is the case in the action of the wick of a lamp or candle, which continues to draw up the oil so long as the liquid is got rid of by combustion from the summit of the wick, but ceases immediately that this process is checked and the capillary tubes remain saturated. So when a bundle of cotton threads is allowed to dip into water, and to hang down over the side of the vessel, a continual dripping takes place at its pendent extremity, simply by the agency of gravity, and the supply is kept up through the agency of capillary attraction; but let the cord be held in a horizontal position, and the overflow ceases.

"To avoid any false application of the phenomena of capillary action to the animal economy, it must be constantly kept in view that a space which is already completely filled with liquid is incapable of exercising any capillary action whatever; that the action of a capillary tube on liquids is due less to the substance of the tube, than to the nature of the liquid with which its internal surface is moistened; and lastly, that it is never by the agency of simple capillarity that liquids are caused to flow forth from the upper end of the tubes into which they are raised." (p. 19.)

Many of the phenomena of capillary action are best studied in that form of it which is commonly termed imbibition, and which consists in the introduction of fluid into porous substances. These may be regarded as channeled out by an immense number of capillary canals, into which the liquid is attracted; and the same law holds good here, as in ordinary capillary action, that the readiness with which the solid substance may be wetted with the liquid, or, in other words, the degree of molecular attraction subsisting between them, is the chief regulator of the result The following experiments, which were performed by Professor Matteucci in conjunction with Professor Cima, appear to us to deserve peculiar attention. Six tubes, each about 8-10ths of an inch in diameter, were filled with finely-sifted and well-dried sand, and their lower ends, having been tied over with cloth to prevent the escape of the sand, were then immersed to the same depth in different liquids, and allowed to remain there for ten hours, care being taken to keep up the surface of the liquids surrounding the tubes to the same height. At first the rise of all the liquids took place rapidly; but its rate soon diminished, and the retardation continued until the stagnation became completed, the point of saturation having been reached. All the saline solutions employed had the same density. The following are some of the results of these comparative experiments, as expressed by the height to which each liquid rose in the cylinder of sand:

| | Millim. |
|---|---|
| Solution of carbonate of soda | 85 |
| sulphate of copper | 75 |
| Serum of blood | 70 |
| Solution of carbonate of ammonia | 62 |
| Distilled water | 60 |
| Solution of common salt | 58 |
| White of egg, diluted with its volume of water | 35 |
| Milk | 55 |

When thick solutions of gum or starch, or fixed oils, were employed, the amount of imbibition was almost nought; and it was but little more when strong saline solutions, or liquids holding finely-divided particles of solid matter in suspension, were employed. This last fact may assist us in explaining why serous infiltrations take place much more readily in the living body, when the density and viscidity of the circulating fluid, and its quantity of solid particles, have been diminished by loss of blood or other causes.

The following experiment demonstrates in a striking manner the degree in which imbibition is affected by the peculiar attractions existing between the solids and liquids employed. Three tubes, respectively filled with sand, pounded glass, and sawdust, were immersed at their lower extremities in water, and three similar tubes in alcohol; the following were the comparative results:

| | Tube with sand. | Tube with pounded glass. | Tube with sawdust. |
|---|---|---|---|
| Alcohol | 85 millim. | 175 millim. | 125 millim. |
| Water | 175 | 182 | 60 |

Thus we see that, whilst the imbibition of the two liquids was nearly the same in the pounded glass, more than twice as much water as alcohol was drawn up by the sand, and more than twice as much alcohol as water was drawn up by the sawdust. Another experiment showed that when the substance was closely pressed in the tube, the amount of imbibition was much increased, as we should expect, from the diminution in the size and the increase in number of the capillary channels, under such circumstances. One of the most remarkable results, however, was obtained by varying the temperature. Two tubes, similarly prepared with sand, were placed in water, the one at a temperature of 59°, and the other at 131°. After 70 seconds, the water had risen 10 millimeters in the former, and only 6 in the latter; but the difference was far more striking after a longer period; for in 11 minutes the water had risen 175 millimeters in the former, and only 12 in the latter. It is impossible not to perceive in this result how much the influence of temperature upon the activity of animal and vegetable life may be explained on simply physical principles. It must be for the mathematician to combine these curious results with those other phenomena of molecular action which have been already alluded to, and which present a series of problems of the highest interest, the complete resolution of which can scarcely yet be anticipated; but the general fact, in its present state—namely, that simple physical imbibition is greatly accelerated by warmth and retarded by cold,—is quite sufficient for the purposes of the physiologist.

The subject must not be dismissed without adverting to the remark made by Professor Matteucci, that the effects of chemical affinity may be developed by the simple play of the capillary forces and of molecular attraction.

"If we reflect that a liquid of any kind constantly elevates itself to the same height in a capillary tube,—that during the imbibition there is always a greater or less production of heat, as the experiments of Pouillet have shown,—that there is, moreover, according to Becquerel, a disengagement of electricity,—and that, lastly, capillary attraction is only exercised at very minute distances, and between the molecules of bodies,—we cannot but admit that this force unites the principal characters of chemical affinity. The observation of Dobereiner's is well known, that if a mixture of water and alcohol be inclosed in a bladder and exposed to the air, the water continually escapes, and the alcohol is retained. In this case, the water is drawn into the membrane more readily than the alcohol, and is dissipated on its exterior by evaporation. Another more conclusive fact is that stated by Berzelius; namely, that salt water, if filtered through a long tube filled with sand, is discharged more or less completely deprived of salt.* I have myself confirmed this experiment, employing for the purpose a tube full of sand about 8 meters (26 feet) in length; and I have found, in fact, that the density of the water issuing from the tube was to that of the liquid introduced by its superior orifice, as ·91 to 1·00. But it must be added, that this difference did not continue in the same degree; at the end of a certain time the saline solution was as dense at its exit from the tube as at its entrance. This proves that the decomposition of the saline solution takes place in the first action of contact between the particles of sand and itself. I have obtained an inverse result, by employing a solution of carbonate of soda, which I caused to traverse a tube full of sand, 10 feet in length. The density of the liquid at its exit from the tube was to that of the liquid as it entered it as 1·005 to 1·000." (p. 29.)

If the solution of saline substances in water is to be regarded as a result of chemical affinity, then unquestionably their separation must be regarded in the same light. But we are not sure that such a mode of viewing the phenomena is correct. The facts themselves, however, are of the highest physiological interest; because they show us how, by a process of simple filtration, an important change may be effected in the proportions of the components of a solution. Thus we see that, in the case mentioned by Berzelius, the saline matter was drawn from the water by the attraction of the particles of sand, until probably every one of them had acquired a thin coating of it and could attract no more; whilst in the second case,

* Is not this the explanation of the frequent occurrence of springs of fresh or brackish water near the sea, in circumstances where collections of fresh water could scarcely be supposed to exist?—REV.

the water appears to have been attracted more powerfully than the carbonate of soda dissolved in it, so that the latter was allowed to pass on, whilst the former was partially retained. We thus see in the ordinary separation of the thin serous fluid, which distends the cavities of areolar tissue, and bedews the surfaces of serous membranes, and which is like a diluted blood-serum, nothing but a process of filtration; which allows the more aqueous part of the liquid to escape from the vessels, whilst the fibrine of the liquor-sanguinis, with a good part of the albumen and saline matter, together with the floating corpuscles, are kept back.

The phenomena of endosmose next claim our attention, constituting, as they do, a group which borders most closely on the domain of purely vital operations. We find Baron Liebig giving utterance to what appears to us a most unjust sneer at physiologists, for their supposed manner of viewing this subject:

> "The commonest phenomena are even yet personified in the minds of many physiologists as peculiar powers, as properties which they are tempted to explain by peculiar causes, differing from other known causes. Thus the restoration of equilibrium between two fluids of different nature, or two solutions of dissimilar substances, separated by an animal membrane, has obtained the names of endosmose and exosmose; and men regard these names as if they were independent things, while the phenomenon is nothing else than a filtration, different from the ordinary one only in so far as that the passage is effected by an attraction (a drawing, an affinity) instead of by pressure." (Animal Chemistry, 3d Ed. p. 165.)

Now we are not aware that the terms endosmose and exosmose have ever been used in any other sense than as the expressions of certain general facts,—namely, as the words import, the existence of an inward and an outward current, under certain conditions. Every one admits that these currents are due to molecular attractions of the same nature with those concerned in the ordinary operations of capillarity and imbibition; but with the alteration in the conditions there is a marked alteration in the results, and physical science has not yet succeeded in fully accounting for the phenomena. These phenomena, however, form a group so distinct, that they may be all conveniently embodied under one general term; and we cannot think of one more appropriate than that which was applied by Dutrochet, who was the first to draw attention to the phenomena. Thus if we say that the form of the blood-corpuscles may be changed by endosmose, we express in a concise way the fact, that if they be placed in pure water or in diluted serum there will be a passage of fluid towards their interior, which will distend and even burst them; whilst if they be placed in a solution of salt or sugar, of greater density than their own contents, the chief current of fluid will take place in the other direction, and the blood-corpuscles will be emptied. Thus the term is used to express the *proximate conditions* of a certain phenomenon, which it brings definitely before the view of the mind. With the *ultimate causes* the physiologist has nothing to do, until physical investigation shall have determined them; which, as we have the authority of Professor Matteucci for asserting, has not yet been effected. For although it might not seem difficult to give a general explanation of the fact, that two liquids of different densities, disposed to a ready admixture with each other (such as syrup or mucilage, and water), should pass towards each other when separated by a porous membrane, and that the more rapid current should be that of the rarer fluid towards the denser,—there are many variations and exceptional phenomena, for which no such general explanation is adequate to account. For instance, when alcohol and water are employed, the principal current or endosmose is from the water towards the alcohol, although the latter is the less dense of the two. A fact still more difficult of explanation, is the agency of sulphuretted hydrogen in immediately checking the process. As soon as the least putrefaction commences in the membranous septum, the endosmose ceases, and the liquid returns by filtration ; and if a fresh membrane be exposed, even for a short time, to sulphuretted hydrogen, no endosmose will take place through it, even between two liquids ordinarily most energetic in their action on one another. In like manner, the introduction of a very small quantity of sulphuretted hydrogen into the liquids employed is sufficient to retard or check the process, even though these liquids, when pure, are powerful supporters of the endosmotic current. And this is probably the reason why the liquids of the large intestine should be an exception to animal fluids in general, in having no endosmotic action with water. One of the most remarkable of all exceptional cases is that of hydrochloric acid. When this liquid has a density of 1·020, the endosmose takes place from the water to the acid ; whilst at the density of 1·015, the chief current takes the contrary direction, being directed from the acid towards the water. But if the temperature of the liquid be raised above 68°, the endosmose again takes place in the first direction.

It was attempted by Dutrochet to determine the relative endosmotic powers of different solutions of the same density ; that is, the relative energy with which they would cause a current of water to pass towards them through the membranous septum. Thus, he states the powers of solutions of gelatine, gum, sugar, and albumen to be to each other respectively as 3, 5, 11, and 17. But these results are true only for one kind of membrane ; for, as the researches of Professor Matteucci have shown, different membranes exercise very different influences over the endosmotic process ; nor is it immaterial which side of the membrane is exposed to the water, and which side to the solution. Professor Matteucci's experiments have been made upon three classes of membranes ; cutaneous, gastric, and vesical. Of the first class he examined the actions in the skin of the frog, the eel, and the torpedo. It was with the skin of the torpedo that he first discovered the important influence effected by the position of the two sides of the membrane. When the external surface of the membrane was in contact with the solution, the rise of the liquid was rapid ; whilst if the internal surface of the membrane were in contact with the solution, the rise of the liquid was very slow in comparison. Thus a solution of gum would rise 30 millimeters, and a solution of sugar even 80 millimeters, in the tube of the first endosmometer ; whilst the same solutions would only rise 18 or 20 degrees, or even much less, in the second. In like manner a solution of albumen would be raised 26 millimeters in the first case, whilst it rose only 13 in the second. The same results were obtained with the skin of the frog. When the skin of the eel was employed, it was noticed that the immediate rise was the same, when a saccharine solution was employed, whichever side of the membrane was turned towards it; but the same inequality soon manifested itself, as in the preceding cases. When a solution of gum or of albumen was employed, the difference was immediately apparent. When the same comparative experiments were tried with alcohol (the passage of water towards which is itself an exceptional phenomenon) still more curious results were obtained. When the skin of the frog was employed, the endosmotic current was found to be most active when the internal surface of the membrane was turned towards the alcohol; but the contrary was the case when the skin of the eel or of the torpedo was used as the septum, the endosmotic current passing most readily from the exterior towards the interior. Some curious results were also obtained on watching the successive changes in the elevation of the column, from hour to hour; from which it appears that the membranes, when no longer fresh, have a different action on the fluids from that which they exert at first. Upon these, however, we cannot dwell; and we shall only stop to notice the following results of a long series of comparative experiments undertaken to determine the relative endosmotic actions of these three membranes upon solutions of sugar, albumen, and gum, and upon alcohol. In each case the *external* surface of the membrane was in contact with the liquid.

| | Skin of eel. | Skin of frog. | Skin of torpedo. |
|---|---|---|---|
| Solution of sugar . . | 15 | 25 | 100 |
| albumen . . | 8 | 15 | 30 |
| gum . . | 6 | 22 | 120 |
| Alcohol . . . | 55 | 80 | 35 |

"This table proves, 1st, that with the skin of the torpedo the endosmotic current is the strongest, if we employ as the interior liquid a solution of sugar, gum, or albumen; 2d, that with these same liquids, the current is much less powerful in the skin of the eel than in that of the frog; 3d, that when alcohol is employed, there is a powerful endosmotic current from the water to the alcohol, which is much stronger than the similar current in the eel, and stronger in this than in the torpedo; 4th, that this powerful current across the skin of the frog takes place in spite of the position of the skin, which is not the one most favorable to the passage of water towards the alcohol; 5th, that the intensity of the endosmose, in the case of any one skin, varies according to the liquids employed; the order, from the highest to the lowest, being as follows:

"*Skin of torpedo.* Solution of gum, syrup, alcohol, solution of albumen.

"*Skin of frog.* Alcohol, solution of sugar, gum, albumen.

"*Skin of eel.* Alcohol, solution of sugar, albumen, gum.

"These last results prove that the order in which Dutrochet arranged these liquids, according to the intensity of the endosmose which takes place between them and water, cannot be regarded as holding good in all cases; we shall see that it cannot even be regarded as invariable in the single case of the membrane of the urinary bladder, which was the one employed by this able experimenter." (p. 51.)

Similar differences presented themselves in the relations of the membranes of the second class to the several fluids; but we shall only notice the influence of the difference of surface in contact with the liquid, in the case of alcohol, where it is very strongly marked. Thus when the membrane of the stomach of the cat had its external surface (i. e. the surface in contact with the muscular coat of that viscus) turned towards the interior of the endosmometer, and consequently placed in contact with the alcohol, the elevation of the fluid in the tube was 22 millimeters; whilst it was no more than 2 millimeters in the contrary position of the membrane. The same disparity showed itself when the membrane of the stomach of the dog was employed; the elevation being as much, in one experiment, as 130 millimeters in the most favorable position of the membrane, and only 6 in the least. The stomach of the lamb gave a similar, but rather less striking result. For most of the other liquids, however, the position of the membrane most favorable to endosmose was that in which its free or mucous surface was turned towards the fluid contained in the endosmometer. But a still more curious variation is presented by the fact that when the lining membrane of the gizzard of the fowl is employed as the septum between water and alcohol, the direction of the endosmotic current is altogether reversed, whatever be the position of the membrane; the flow being now invariably from the alcohol towards the water, but being more intense when the internal or free mucous surface was in contact with the alcohol. Of this remarkable fact Professor Matteucci states that he has fully satisfied himself by repeated and careful experiments.

When the membrane of the urinary bladder of the ox was employed, in a fresh state, with a solution of sugar, it was found that the endosmotic current took place most readily from the attached towards the free surface of the membrane; whilst the contrary was the case, when a solution of gum was substituted for the sugar. With the gummy fluid the following curious phenomenon was further observed: that, during the first hour of the experiment, the fluids frequently fell in the endosmometers, though they subsequently rose much above their original elevation. When a solution of albumen was employed, it was found that *no* endosmose took place through the fresh mucous membrane of the urinary bladder, when pure water was on the other side of it. All these curious differences vanish, however, or undergo great modifications, when, instead of fresh membranes, those which have been dried, or which have undergone more or less alteration by putrefaction, are employed. It is evident that the method of experimenting adopted by Professor Matteucci greatly increases the value of his results to the physiologist; since it gives us the nearest possible representation of the operations of *living* membranes under similar circumstances.

It appears, from other experiments devised by Professors Matteucci and Cima, that the elevation of the fluid in the endosmometer cannot be regarded (as Dutrochet supposed that it might) as an absolute measure of the difference between the stronger or endosmotic and the weaker or exosmotic current. Two endosmometers were prepared in the usual way, with the skin of the frog or of the eel; the external surface being directed towards the contained fluid in one instrument, and towards the water in the other. The endosmometer was then filled with a solution of salt, which allowed the subsequent changes to be more readily determined than could have been effected with a solution of gum or albumen, and was immersed in its own bulk of distilled water. After the experiment had continued for some hours, it was found that (as in previous instances) the elevation of the fluid was greatest in that endosmometer in which the internal surface of the skin was in contact with the water. It might be expected that the saline solution in this endosmometer, being now of greater bulk than that contained in the other, would have experienced more dilution, and be found to be of inferior density. But the contrary was the case; for the saline solution which stood the highest in the endosmometer was found to have the greatest density; whilst the water in which it had been immersed, and of which the diminution in bulk was the greatest, contained a quantity of saline matter much less than that which surrounded the other endosmometer, where the interchange appeared to have been much less. Thus it became evident that the elevation of the liquid in the tube of the endosmometer cannot be regarded as at all indicative of the positive energy of either current; and we agree with Professor Matteucci in thinking that the results of the experiments already cited, in regard to the different influence of the two sides of the membranes employed, seem best explained on the hypothesis that the endosmotic current is the same, or nearly the same in the two positions of the membrane, whilst it is the exosmose which chiefly varies, being weakest in the endosmometer which presents the highest elevation, and *vice versa.*

We think that our readers will agree with us as to the physiological importance of these facts; since it is impossible but that they should have a definite and constant relation with the phenomena of the living body. No one can be more sensible, however, than Professor Matteucci himself appears to be that the subject is at present but very imperfectly elucidated; and that a vast number of new experiments are required, to develope the laws of the process, and the modifications induced in it by the variations in the properties of the several membranes of the body in different animals. That the endosmotic properties of these membranes have a relation to the purpose they have to serve in the living economy is sufficiently obvious from the following considerations:

"The exosmose of solutions of gum, sugar, and albumen, towards water, takes place most readily from the internal to the external surface in all the cutaneous membranes we have examined. It is precisely in this direction that an abundant mucous secretion traverses the skin of the frog, the eel, the torpedo, and other animals. The endosmose of water towards these solutions is less considerable from the external to the internal surface of the skin, than when it takes place in the contrary direction. Consequently, if we do not admit that we may entirely explain on this principle the direction of the mucous secretion, and the feeble absorption of the water in which these animals live,—functions which must always bear a certain relation to one another to take place with regularity,—at least it cannot be denied that these physical conditions are favorable to them. It is evident this function of the skin would be executed imperfectly, or would cease altogether, if, in animals which constantly inhabit water, this membrane acted by endosmose in a direction opposed to that in which we have found it to operate most powerfully.

"We may pass by the opposite result which is shown, when water and alcohol act on one another through the skin of the frôg. Alcohol is a liquid which has no analogy among those which are found in the bodies of animals; and the anomalies which we have met with in employing it as an endosmometric liquid, if they be true also of the skin of man, would find their application rather in therapeutics than in physiology.

"The constancy observed in regard to the most favorable direction for endosmose and exosmose through the skin, is no longer experienced when we employ the mucous membranes of the stomach of different animals. But no one is ignorant how complicated is the function of the stomach; nor that of the substances which are introduced into the organ, some are not absorbed at all, and others very slightly. Moreover, we again repeat that this subject particularly requires being elucidated by new researches. When we remark that the direction most favorable for endosmose, between water and solution of sugar, for example, is not the same for the stomach of a ruminant and for that of a carnivorous animal, it is clearly demonstrated by this fact that the phenomenon of endosmose is intimately connected with those essential modifications with which the digestive functions are intimately connected in these two orders of animals." (p. 69.)

"I must not conclude without citing to you the recent experiments of Poisseuille, with a view of explaining by endosmose the purgative action of certain saline substances. He found that endosmose took place through the animal tissues from the serum of the blood towards Seidlitz water, solutions of sulphate of soda, common salt, &c. This is precisely what occurs when these medicines are administered internally. The excrements contain an abundant and unusual amount of albumen; and we can scarcely help admitting that endosmose takes place from the serum of the blood to the saline solution introduced into the intestinal tube, through the walls of the capillary vessels of the latter. But to remove all doubt of the justice of this application of the doctrines of endosmose by Poisseuille, it was necessary to demonstrate that endosmose would continue when one of the liquids is in motion, and is continually being renewed. This has been recently proved by Dr. Bachetti, who has shown that the rapidity of the endosmose is considerably augmented when one of the liquids is in this state of continual renewal. This result, moreover, is in accordance with the principles of the theory of endosmose; for the exchange of liquids constantly taking place through the membrane certainly tends as it proceeds to diminish the force of endosmose; or, in other words, the conditions which produce the phenomenon are maintained so much the better, as the liquids remain longer without admixture. Poisseuille also has shown that endosmose ceases to take place through a membrane, after a certain period of activity; but that the property may be excited anew by the agency of other liquids. The most remarkable fact discovered by Poisseuille, is that of the influence exerted by muriate of morphia. This substance, added to saline solutions, considerably weakens the endosmose of the serum towards the solution, and finishes by changing the direction of the current. This fact has been confirmed by Dr. Bachetti." (p. 71.)

How, asks Professor Matteucci, can such a fact as this be overlooked, in the explanation of the agency of morphine and of the preparations of opium in checking diarrhœa and producing constipation? And what a new field of inquiry is opened up to us, when it is shown that these supposed ultimate facts in the physiological action of medicines are so clearly to be referred to physical conditions!

In the application of these principles to the phenomena of absorption in animals and plants, which occupies the fourth lecture, we do not find much that need detain us; since it must be at once obvious to those acquainted with those phenomena, how large a part of them may be referred to physical laws. And it is further apparent, that a proper knowledge of these laws would have prevented the diffusion of many grave physiological errors; such, for example, as the exclusive absorbent power of the lymphatics and lacteals. For the permeability of the animal tissues once being demonstrated, and the influence of the movement of fluid through membranous tubes in attracting towards them fluids external to those tubes having been established, it follows as a matter of course that the sanguiferous as well as the absorbent system must participate in the imbibition of fluid; and that the venous system will be much more concerned in it than the arterial, on account of its more superficial position, the greater extent of surface of its vessels, the comparative thinness of their parietes, and the inferior pressure of the circulating fluid against their walls. The permeability of the animal tissues *en masse* is readily established by such an experiment as the following. If a frog recently dead have its hind legs only immersed for some hours in a solution of prussiate of potass, and it be then cut into fragments, it will be found that if any part of its body be touched with a rod dipped in a solution of sulphate of iron, a blue spot will immediately be formed; showing the universal diffusion of the prussiate of potass through its tissues. If the same experiment be tried upon a living frog, the same result will be obtained. If the immersion be continued, however, for a short time only, there will be found but little indication of the prussiate of potass in the muscular masses of the limbs, whilst it is met with abundantly in the heart and lungs; showing that, in the living animal, the imbibition and diffusion take place most speedily through the circulating apparatus.—The influence of the movement of the fluid within the vessels is shown by the following experiment. If we take a long portion of one of the principal veins of some large animal, and fix it by one extremity to a tube proceeding from an opening at the bottom of a vessel filled with water, whilst we attach a stopcock to the other extremity and if, with this stopcock closed, we immerse the surface of the vein (which is distended with water from the reservoir) in water acidulated with sulphuric or hydrochloric acid, it is some time before the liquid in the reservoir presents any traces of the acid. But if, instead of waiting for the appearance of the acid in the reservoir, we open the stopcock, and allow the water to discharge itself, we immediately discover the presence of the acid in the liquid which has thus been made to flow through the vein. The same result would present itself with a tube of clay or other inorganic substance; the phenomenon being one of a purely physical character.

Professor Matteucci is far from maintaining that *all* the phenomena of the introduction of fluids into the tissues of animals and plants can be explained upon physical principles; and he does not appear to us to have any clear idea as to the point at which the line is to be drawn. For ourselves we are disposed to think that a very large part, if not the whole, of the phenomena of vegetable absorption and of the ascent of the sap may be thus accounted for, the conditions under which they occur being duly kept in view. The vegetable physiologist is well aware how completely the continuance of active absorption by the roots is due to the exhalation of fluid by the leaves; the former process being brought to a stand almost immediately that the latter is checked, and being found to recommence immediately upon the renewal of the latter. Hence it would appear that the rise of the sap in the stem has the same kind of dependence upon the dissipation of the greater part of its fluid through the agency of the leaves, that the rise of the oil in the wick of a lamp has upon the combustive process taking place at its summit. And we find, moreover, that this ascent continues through the upper part of the stem and branches, when they are severed from the trunk and roots, and have the cut surface immersed in water; which shows that the continued ascent is not dependent on any *vis a tergo* in the roots, but on a *vis a fronte* in the leaves. But, on the other hand, if the roots be immersed in water, a continued discharge of fluid will take place for some hours through the cut surface of the stem in which they terminate ; showing that the absorption continues through their spongioles. We are disposed to think with Dutrochet that this is a pure act of endosmose ; and it seems to us that the admixture of the viscid elaborated sap with the crude fluid just absorbed, which is known to take place in the roots, is the very condition required for maintaining the density of the fluid within the tissues of the plant, at that point above the density of the fluid in the soil around, which is a necessary condition of endosmose. If such be the fact, it certainly accounts for the cessation of the absorption by the roots, within a certain period after the removal of the upper part of the stem and branches ; for when these have been separated, there will no longer be the requisite supply of descending sap, to serve to uphold the density of the contents of the vessels of the roots ; the fluids which they contain will be gradually diluted until they are little else than pure water, and the endosmose will cease. All the phenomena of absorption in animals appear to us distinctly referrible either to physical principles or to the laws of cell-growth. The former will sufficiently account for the entrance of liquids and of soluble substances into the tubes of the absorbent and sanguiferous system ; whilst the latter seems concerned, as Mr. Goodsir has shown, in the special selective agency of the absorbent vessels.

Professor Matteucci's fifth lecture gives a succinct and accurate account of the present state of our knowledge of the function of digestion ; which no well-informed physiologist has any longer the least hesitation in regarding as a process whose explanation upon physical principles is nearly complete, and whose entire elucidation must be looked for in a further application of the same principles. As we have at various times brought this subject under the consideration of our readers, we shall confine ourselves at present to the notice of a few points of novel interest, which recent observations have contributed to settle. The solution of the albuminous or proteine compounds in the stomach, by the conjoint action of a free acid, and of an animal compound to which the name of pepsine has been given, has long been an established fact ; although doubts still remain as to the nature of the acid, and the precise nature of the organic compound needs further elucidation. A most important fact has been ascertained, however, by MM. Bernard and Barreswill, namely, that the organic matter of the saliva and of the pancreatic juice (which secretions are normally alkaline) has the same solvent power for albuminous matters, when acidulated, as that which is possessed by the gastric secretion. The solution of albuminous matters being complete, these substances will necessarily be introduced by simple physical absorption into the blood-vessels so copiously distributed upon the walls of the stomach and intestinal canal ; and the experiments of MM. Tiedemann and Gmelin, which have been recently confirmed by MM. Bouchardat and Sandras, leave it doubtful whether the albuminous matters found in the lacteals are ever taken up directly from the contents of the intestinal canal, or whether they are not derived indirectly from the surrounding blood-vessels. The relative position of the blood-vessels and lacteals in the villi,—the latter being completely surrounded by the former,—would seem to add support to this view; and it further seems to correspond well with what we know of the general functions of the absorbent system, which may be looked upon as a great glandular apparatus, its elements being dispersed over the entire body, destined to be continually removing albuminous matter from the blood, and to be restoring it again in the form of fibrine; the absorbents of the intestines having the additional function of introducing fatty matter, in the peculiar form presently to be adverted to.—The experiments of MM. Bernard and Barreswill further show, that the peculiar organic matter of the saliva and pancreatic fluid, when accompanied with an alkali (as in the normal condition of those secretions), has the power of transforming starch into sugar. Thus it would appear that the digestion of amylaceous substances commences in the mouth, during the processes of mastication and insalivation; that it is suspended during their continuance in the stomach, the acid condition of the gastric fluid being incompatible with it; and that it is renewed when the pancreatic secretion is poured into the intestinal canal, being (it would appear) the special object which that secretion is destined to perform. Various recent experiments have thrown light upon the way in which the saccharine matter thus formed is afterwards disposed of. From the researches of Drs. Buchanan and Thomson it would appear that a certain amount of it may be detected in blood drawn shortly after a full meal; but that it soon disappears, being probably burned off by the respiratory process almost as fast as it is introduced. The inquiries of MM. Bouchardat and Sandras make it probable, however, that the greater part of the sugar and dextrine produced by the metamorphosis of starch is converted into lactic acid during its passage along the alimentary canal; and that it is absorbed, and applied to the maintenance of the respiratory process, in this form. If pure sugar be introduced in any large quantity into the blood-vessels of a healthy animal, it is speedily eliminated by the urine, and is thus altogether inert as a supporter of the combustive process; and it is probably to avoid its too rapid absorption, that the production of saccharine from amylaceous matter takes place so slowly in the alimentary canal. The long-disputed question as to the origin of fat, which seemed to have been finally settled in favour of the view upheld by Liebig,—their fatty matter may be produced from amylaceous or saccharine compounds,—now appears likely to be completely resolved by the discovery of the mode in which this transformation is effected; namely, by the agency of the bile. From the recent experiments of H. Meckel it appears, that if bile be mingled with grape-sugar, and be kept for some time at a warm temperature, a much larger quantity of fatty matter is to be found in the mixture, than could have existed in the bile. Thus in one experiment, the amount of fat in an equal quantity of the same bile having been found to be from 48 to 54 grammes, the amount contained in the mixture of bile and grape-sugar after five hours was ·87 grammes, and after twenty-four hours was 1·84 grammes.

On the mode in which fatty matters are introduced into the system, Professor Matteucci affords us some valuable information from his own researches. Physiologists are well aware that this introduction takes place chiefly, if not entirely, through the medium of the lacteals; and the experiments of Schwann have rendered it next to certain, that one part of the functions of the bile is to reduce the fatty matters of the ingesta to a condition fit for absorption. When an animal has been fed on substances in which oily matter exists, the chyle is always found to contain fatty particles, some of which are solitary and of appreciable size, varying in diameter from 1-25,000th to 1-2000th of an inch; whilst others, of dimensions more minute than even the smallest of the preceding, are crowded together in the fluid of the rich milky chyle, and give to it its opalescent character. That these last particles, which form what has been termed by Mr. Gulliver the "molecular base" of the chyle, are chiefly of a fatty nature, is shown by their ready solubility in ether; the addition of which to the chyle causes the whole molecular base instantly to disappear. There is no evidence that any fatty matter exists in the chyle in a state of perfect solution; and as we know that oily substances in general are not capable of endosmotic action, especially when the membrane is moistened with an aqueous fluid, there is a difficulty in understanding how the fatty particles can find their way from the intestinal canal into the lacteal vessels. This difficulty has been in great part resolved by the experiments of Professor Matteucci. He has shown that if water be rendered very slightly alkaline, so as scarcely to act upon test paper, and some oil be then shaken up with it at a temperature of from 95° to 104°, an emulsion is at once formed, which has very much the aspect of milk. The liquid when left to itself, shows a further analogy to milk; for it separates into two layers, of which the upper one is the more opaque, and contains oily particles of appreciable size, whilst the inferior one, though less opaque, still preserves the milky aspect, and has innumerable minute particles of an oily character diffused through it. Thus we see that a very small quantity of alkali, such as is contained in the biliary and pancreatic secretions, though it will not dissolve the oily matter, reduces it to precisely that state of fine division in which we find it in the chyle. The following experiments show that in this condition it may be made to pass through an animal membrane.

"Having filled a portion of intestine with this emulsion, I plunged it into the alkaline solution just described, keeping the temperature at from 95° to 104°. After a certain lapse of time, the latter became turbid, and assumed the characters of the emulsion within; so that it may be fairly concluded that some of the latter had escaped through the membrane, and diffused itself through the liquid around.—The following experiment appears still more conclusive. I filled an endosmometer with a very weak alkaline solution, and immersed it in the emulsion which I have shown you. The membrane employed was, as usual, that of the urinary bladder of the ox; and the two liquids were at the temperature of 86° at the commencement of the experiment. Endosmose took place, and the emulsion penetrated the alkaline solution, so as to raise the column of liquid 30 millimeters in a very short time." (p. 105.)

Thus we see how the slightly alkaline condition of the liquor sanguinis and of the chyle will operate physically in promoting the entrance of the fatty particles; since for their passage through an animal membrane nothing more is required, than that they should be reduced to a state of very fine division (which is accomplished by the admixture of the biliary and pancreatic secretions), and that on the other side of the membrane a slightly alkaline fluid should exist. And it is to be remembered here, as elsewhere, that the constant motion of the blood compensates for the weakness of its chemical actions; a much more powerful effect being produced by a very weak solution in rapid movement through the vessels, and therefore continually renewed, than by a much stronger one that remains always at rest.

We may pass over the sixth and seventh lectures, which treat of Respiration, Nutrition, and Animal Heat, as not containing any original observations of importance. The physics of these departments of physiology are better understood, and more fully treated in physiological writings, than those of most others. We may stop, however, to notice the valuable discovery made not long since by Valentin and Brunner, that the quantities of oxygen absorbed, and of carbonic acid exhaled in the respiratory process bear a constant relation to each other; and that this relation corresponds so closely with their mutual diffusion-volumes, as determined by the law discovered some years since by Professor Graham, that no reasonable doubt can remain, that the interchange takes place in strict accordance with physical principles. There will always be an excess of oxygen above the carbonic exhaled; the proportion being 1174 parts of the former to 1000 of the latter; and as carbonic acid contains its own bulk of oxygen, there will always be a positive surplus of the latter, amounting to 174 parts in every 1174 absorbed, to be employed in the system for other purposes than the generation of carbonic acid. These other purposes seem to be principally the union with hydrogen, forming part of the water which is exhaled; and the combination with the sulphur and phosphorus introduced with the proteine-compounds, which are excreted in the condition of sulphuric and phosphoric acids. These additional combustive processes, with others, perhaps, still to be discovered, seem to leave no reasonable difficulty in accounting on purely chemical principles for the entire amount of caloric generated in the living animal body.

In the eighth lecture, on the Phosphorescence of organized beings, Professor Matteucci gives the results of several original experiments upon the luminosity of the glow-worm, and the conditions under which this interesting phenomenon occurs. He fully confirms all that had been previously advanced by Macaire, in regard to the effect of oxygen in brightening the light, and of carbonic acid in extinguishing it; and he has shown that the same result happens when the luminous segments separated from the bodies of the animals are exposed to these gases respectively. An increase of energy in the light is produced also in the dead as in the living state, by a high temperature. When several worms are placed in a limited amount of air for some time, the light gradually becomes more dim and is at last extinguished; and on examining the air it is found that the oxygen has nearly disappeared, and is replaced by carbonic acid. If the separated luminous segments alone be placed in a limited quantity of air over mercury, a similar change is produced, though with less rapidity; an appreciable quantity of carbonic acid being generated so long as the luminosity continues. There can be no doubt, therefore, that the phosphorescence is due to a combustive process, taking place in the peculiar substance which is found beneath the integument of the luminous segments. Of this substance, enough has been obtained by Professor Matteucci in a separate state, to be made the subject of chemical examination. The following he states to be its physical and chemical characters. It has a granular aspect and a yellowish colour; and is traversed by great numbers of tracheæ. It possesses a peculiar odour, somewhat resembling that of the perspiration of the feet; it is neither acid nor alkaline; it desiccates quickly in air; it undergoes a sort of coagulation when treated with dilute acids; it is not soluble in alcohol, ether, or weak alkaline solutions; it is dissolved and changed by concentrated sulphuric and hydrochloric acids, with the aid of heat; but the solution made in this manner does not become blue, which excludes the idea of the presence of albumen. When it is heated in a tube, the ordinary ammoniacal products are set free. Professor Matteucci has several times carefully tested it for the presence of phosphorus, by calcining it with nitre, and treating the dissolved residue with reagents which would indicate the presence of phosphates; but always without success. It hence appears that the luminous matter is a substance *sui generis*, but made up of the usual components of the animal tissues, namely oxygen, hydrogen, carbon, and nitrogen; and there is the less difficulty, therefore, in admitting and accounting for the instances of luminosity in the living human subject, to which Sir Henry Marsh a few years ago directed our attention. (See Prov. Med. Journ. 1842). In these and similar cases, the luminosity was probably due to the production of the luminous matter from the food or from the disintegrating tissues, by a process of chemical transformation which is normal in the luminous insect, though abnormal in man. There is no want of instances of the occurrence of slow combustion attended with luminosity, in organized matters of various kinds, such as decaying wood, greasy cotton, and powdered charcoal. The variations in the light which occur in the living insect, the greater brilliancy which it exhibits when irritated, and the occasional entire extinction of it for a time, appear to be fully accounted for by the control which the animal possesses over the amount of atmospheric air that passes through the tracheæ of the luminous matter. When the spiracles are shut, and no air enters, the light is extinguished; on the other hand, when the respiration is accelerated, more oxygen than usual traverses the air-tubes of the luminous matter, and its brightness is increased.

We now come to those departments of physico-physiological inquiry, in which Professor Matteucci has earned his highest reputation; those namely, which relate to the Electrical phenomena of Living Beings. And as we have not on any former occasion brought these formally under the consideration of our readers, we shall avail ourselves of the present opportunity of doing so. The ninth lecture treats of the muscular current of electricity, our knowledge of which (except in the special case of the frog, to be presently noticed) is entirely due to the inquiries of Professor Matteucci. In order to understand the nature of the experiments to which we shall presently refer, our readers must be first made acquainted with the peculiar indicator of the existence of electric currents, which Professor Matteucci has found most useful. This is nothing else than the leg of a frog recently killed, separated from the body, with the crural nerve dissected out from its junction with the lumbar plexus, and detached from the muscular mass of the thigh, which is altogether removed; the leg, with this nervous cord hanging freely from it, is inclosed in a tube of glass covered with a non-conducting varnish; and the apparatus now forms a galvanoscope peculiarly fitted to determine the existence of weak electric currents. For if, holding the tube in the hand, we cause the nerve hanging freely from its extremity to touch any body whose electric state we wish to determine, at two points, the existence of even a very feeble electric current between those points will be at once indicated by the contraction of the muscles of the leg. Now if we make an incision into any muscle of a living animal, and insert into the wound the nerve of this galvanoscopic frog, in such a manner that it shall touch both the deep and superficial portions of the wound, we immediately find that muscular contractions are excited in the frog's leg; showing the existence of a current between the different parts of the muscle. This phenomena will manifest itself in the muscles of any animal whatever; and not only in those of the living animal, but in those of animals recently killed, or in muscles lately separated from the body; the current ceasing, however, more speedily in the muscles of warm-blooded animals, than in those of reptiles and fishes.

In order to discover the direction and the intensity of this current, and the conditions under which it presents itself, it is necessary to make use of the ordinary galvanometer; but the employment of this instrument requires many precautions, to ensure freedom from error; which are detailed by Professor Matteucci in his work on the Electro-physiological Phenomena of Animals. By means of this instrument, carefully employed, it may be proved that the current *constantly* proceeds from the internal part towards the external surface of the muscle; and having obtained this knowledge, Professor Matteucci next proceeds to construct a *pile* of muscles, by which the current may be rendered more intense, and its conditions better determined. This pile may be composed of the thighs of frogs cut across, and so connected one with the other, that the external surface of each shall be in contact with the internal surface of the next; or it may be made up of isolated muscles of any other animal, the same precaution being employed. In this manner the intensity of the current is increased, in precise accordance with the augmentation in the number of single portions employed; and a considerable deflection of the galvanometer is the result. No such deviation takes place when the pile is composed of other animal tissues and organs, such as membranes, nerves, the brain, liver, lungs, &c.; the signs of electric disturbance obtained from such being of the feeblest character. The muscular current varies in intensity according to the activity of the nutrition of the muscle. It is at first more powerful in warm- than in cold-blooded animals; though it ceases much earlier. In cold-blooded animals it is very much weakened by previous exposure of their bodies to a low temperature; but this has not the same effect upon warm-blooded animals, on account of their independent calorification. It is remarkable that whilst the muscular current is but little affected when the animals are poisoned by narcotics, by carbonic or hydrocyanic acids, or by arseniuretted hydrogen, it should be almost completely destroyed by sulphuretted hydrogen; both warm- and cold-blooded animals, asphyxiated by this gas, losing nearly all power of manifesting it. In some of Matteucci's experiments with the muscular pile, the successive portions of the frog's thighs were not brought into direct contact, but the communication was established by means of the trunk of the crural nerve dissected out; this did not make any difference in the results, except that the current was weakened by the imperfect conducting power of the nerve. That the nerve in this case acted simply as a conductor was shown by the fact, that the result was the same, in whichever direction the current was made to pass through it; and that a thread of cotton moistened with distilled water answers the purpose equally well. Other experiments demonstrate that this muscular current is altogether independent of the nervous system, and that it must be produced by the molecular changes which are continually occurring in the muscle itself. The remarkable influence of sulphuretted hydrogen seems readily explicable on this view; since the powerful effect of this gas in checking endosmotic action would bring those molecular changes to an immediate stand.

In the tenth lecture we find an exposition of the interesting phenomena of the Electric Fishes, and of the *courant propre* or (supposed) peculiar current of the frog. We shall not follow our author through his account of the former subject, luminous and concise as it is; but must content ourselves with adverting to those points which have been established by his own researches, and which have the most immediate bearing upon the theory of this remarkable class of phenomena. It is easily shown that all the phenomena of the discharge of the Torpedo (upon which, from the comparative readiness with which it may be obtained, many more researches have been made than upon the Gymnotus) are to be attributed to an electric current generated by the instrumentality of the electric organs. Each surface of these organs possesses a different electrical state; the dorsal surface being positive, whilst the abdominal is negative. Now if the electric organs of a lively torpedo be completely separated from the body of the animal, leaving the nervous trunks undivided, and the surfaces of the organs be covered with prepared frogs, and the conducting wires of a galvanometer be also applied to them, it will be observed, on irritating the nerves, that the frogs and the galvanometer indicate the existence of a current in the electric organ; the manifestations of the current being seen over the whole of the organ if all its nerves are irritated, but being confined to that part of it to which the individual nerve is distributed, if only one of the nerves be affected. There is no difficulty in proving, that the exciting cause of the discharge is an influence conveyed through the nerves from the encephalon. These nerves have their centre in a pair of lobes or ganglia quite distinct from the ganglia of which the fish's encephalon is ordinarily composed; and whilst the irritation or the removal of the latter produce no effect upon the electric powers, the slightest irritation of the electric lobe on either side produces violent discharges in the corresponding electrical organ, whilst the destruction or removal of that lobe totally and permanently destroys the electric power. But it does not hence follow, as some have supposed, that the electricity is generated in the brain, and that the electric organ is simply the instrument of its manifestation. Such an idea is manifestly contrary to all *a priori* probability; for whilst we have, on the one hand, a large and complex apparatus, evidently ministering to the peculiar powers of the electric fishes, being superadded in them and in them alone to the ordinary elements of their system, we find no such enlargement of the nervous centres as would at all warrant the idea that the latter are the real generators of the electricity. The fact appears to be, that the relation of the nervous system to the electric apparatus is essentially the same as that which it bears to the muscular; namely, that it is the means by which the independent powers of the latter are called into activity. We shall not now recapitulate the proofs, to which we have referred on various occasions, of the existence of the property of contractility as an endowment essentially belonging to muscular fibre, to which innervation is the most direct and powerful stimulus; but we shall content ourselves with remarking that Professor Matteucci's experiments furnish evidence of the same kind, with regard to the generation of electricity in the electric organs of the torpedo &c., and the agency of the nervous system in calling forth its manifestations, rather than in directly occasioning its production.

The structure of the electric organ is very simple and uniform. It is entirely composed of a series of hexagonal prisms of membrane, strongly resembling a honeycomb in their arrangement. Each of these prisms is divided transversely by a number of membranous partitions; so that the whole prism is in fact composed of a pile of flat hexagonal chambers. These chambers are filled with a fluid, which consists of water holding in solution about one tenth part of albumen, and a little common salt; and upon their walls are distributed the nervous filaments proceeding from the electrical lobe. These filaments are believed by Professor Savi (the results of whose anatomical researches are adopted by Matteucci) to terminate in loops both at their central and their peripheral extremities. Now if a portion of any one of these prisms be removed from a living animal, and the nerve of a galvanoscopic frog be brought into contact with it, any mechanical irritation of the fragment, even though it be no larger than a pin's head, will suffice to produce contractions in the frog's leg. When it is remembered that the number of these prisms, in a single organ of the torpedo, was ascertained by John Hunter to be four hundred and seventy, we need not be surprised at the large amount of electricity generated by a mass of which so small a portion can exert a sensible effect. That the electricity is not conveyed by the nerves to the electric organ further appears from the fact, that the discharge is prevented from taking place by *tying* their trunks, as completely as it is by *dividing* them; the former of which operations makes no difference in their power of conducting electricity. In this as in other respects, there is a close analogy between the agency of the nerves on the electric organ, and their influence on muscles. It is important, in any theory we may form of the mode in which the electric disturbance is produced, to bear in mind that the two ends of the prisms are the points whose electric conditions are most opposed to each other. These extremities correspond with the two surfaces of the body in the torpedo; but in the gymnotus, in which the electric organ extends through a large part of the length of the animal, the direction of the prisms is the same; and consequently the parts between which the discharge should be the most powerful are the head and tail, which is exactly the case, the fish always endeavouring to apply these parts to any body through which it is about to discharge itself. Professor Matteucci suggests that the influence of innervation may be exerted simply in separating the two electricities ordinarily balanced in each prism, so that the positive shall be driven to one extremity, and the negative to the other; and he adduces as parallel cases, the agency of heat on the tourmaline and on some crystalline metals, the effects of chemical action, friction, compression, &c. He explains, on this hypothesis, the independent action of a fragment of one of the prisms, by supposing that the irritation acts primarily upon the nervous filaments it may contain. Altogether, the immediate cause of the electric disturbance remains still obscure; but it is something to know where to look for it, and to find it removed from that convenient repository of causes of all unexplained phenomena,—the nervous system.

The effects of the (supposed) peculiar electric current of the frog were first observed by Galvani; who found that a frog prepared in the ordinary manner would execute muscular contractions, when the lumbar nerves were brought into contact with the muscles of the thigh or of the leg. The existence of such a current, however, circulating from the legs to the body of the animal, was first shown with the aid of the galvanometer by Nobili, who made numerous experiments upon it. Its characters at first appeared sufficiently distinctive to induce Professor Matteucci still to rank it in a separate category, after the discovery of the general muscular current which is common to all animals; but he has been recently led, by several considerations, to regard it as a peculiar form of the latter. He found that its intensity was affected by precisely the same conditions as those which affect the muscular current; and that it might be explained by a due attention to the relative arrangement of the muscular and tendinous structures. When a thick muscle terminates in a slender tendon, this last, being in connexion with the interior or deep-seated parts of the muscle, corresponds with these in its electric condition; and it is consequently in an electric condition opposed to that of the surface of the muscle. This may be shown by examination of muscles of similar character in other animals; such as the pectorales of birds. Now the relative arrangement of the muscles, tendons, aponeuroses, &c. in the frog especially favours the peculiar manifestation of the muscular current which has been just described; and this animal is no longer to be regarded in the exceptional light in which it was long viewed by electricians.

In the eleventh lecture, on the Physiological Action of Gravity, of Light, and of Heat, we find nothing deserving of notice, save the extreme meagerness of the treatment of these important subjects. A few well-known facts are brought together; but nothing like a general view of the operations of these agents on the living body, whether in plants or animals, is attempted. In regard to heat, whose agency is so essential a condition of all vital action, the deficiency is peculiarly striking, when contrasted with the amplification of all that relates to electrical phenomena. As the professor has taken other opportunities of bringing before the public his original researches on the latter subject, we do not think that he has done wisely in making them form so prominent a portion of the present course, to the exclusion of other topics of at least equal importance. The twelfth and thirteenth lectures are devoted to an exposition of the Physiological Action of the Electric Current; and form a complete resumé of all that is certainly known on this subject. Of this we shall present our readers with a concise view; to serve as a guide whereby their therapeutic applications of this agent may be directed.

In the first place Professor Matteucci lays it down, that no effect can be produced, save by the electric *current;* and that all the supposed results obtained by *static* electricity,—as when a plant or animal is insulated and charged with electricity by the electric machine,—are fallacious. Although this statement may be perfectly true as regards the inertness of static electricity, yet it does not follow that a plant or animal thus electrized should be uninfluenced by this agent; since we apprehend that the constant radiation of electricity from the numerous points of its surface must give rise to a condition that shall correspond with the passage of a current through it. Putting aside this question, however, we proceed to consider the results of the *dynamic* form of electricity, or the electric current. If we place the two conducting wires of a galvanic pile in contact with two points of a nervous trunk of mixed endowments, in a living animal, that part of the trunk being carefully insulated, we find, on closing the circuit, that powerful muscular contractions are induced, whilst at the same time the animal gives signs of pain. Whilst the circuit is closed, however, these phenomena no longer manifest themselves; but they are renewed when it is interrupted, ceasing again in a few moments. They are again renewed when the circuit is again closed; and this whether the current be transmitted in the *direct* course, i. e. from the roots of the nerve towards its periphery, or *inversely* from the periphery towards the centre. It is observed, however, that the movements are most energetic when the direct current is first transmitted; whilst the signs of pain are the greatest when the inverse current is employed. If the current, instead of being transmitted along the nerve, is passed *across* it, scarcely any effect is produced. After experimenting for some time upon the same animal, we find that all these phenomena cease to present themselves, the animal no longer giving any sign of the passage of the current; the period required to exhaust its influence will be shorter as the current is more powerful. If the animal be then allowed to remain for some time at rest, or the current be rendered more powerful, the original phenomena are reproduced. Now if we study the phenomena which present themselves when the manifestations of the current are becoming weakened, but are not yet entirely ceased, it will be observed that when the direct current is interrupted, the contractions of the inferior muscles (that is, of the muscles supplied by the part of the nerve below that at which the current is applied) are feeble; whilst those of the muscles of the back, as well as the agitation and cries of the animal, remain nearly as before. On the other hand, when the current is renewed, it only produces contraction of the inferior muscles. Precisely the opposite is the case in regard to the inverse current; for the contractions of the muscles of the back, the movements of the ears, and the cries, only occur at the moment when the circuit is closed, scarcely any contraction being seen in the inferior muscles; whilst the latter are alone thrown into contraction when the circuit is closed.

"We may then divide into two different periods the action of the electric current which excites the nerves of a living animal. In the first, the irritation of the nerve is transmitted in all directions, towards its central part as well as towards its periphery, both at the commencement and the conclusion of its passage; and this independently of the direction of the current. But in the second period, the excitation of the nerve is transmitted in the peripheral direction alone, at the commencement of the passage of the direct current, and at the instant when the inverse is interrupted; whilst, on the contrary, the irritation of the nerve is transmitted towards its centre, when the direct current is interrupted, or the inverse one is closed. These results may be expressed more simply by the general statement, that the current acts in the direction of its own course when the circuit is closed, and in the contrary direction when it is interrupted." (p. 232.)

It is easily shown that the muscular contractions thus excited by the electric current are of two kinds: those of the muscles supplied by the nerve below the point of excitation being *direct*, whilst those of all other muscles are *reflex*, being excited through the medium of the spinal cord, to which the irritation is conveyed.

When similar experiments are made upon an animal recently killed, it is found that a very feeble galvanic combination acts only according to the above law; whilst, if a more powerful one be employed, the contractions at first manifest themselves in all the muscles both at the closure and at the interruption of the circuit, in whichever direction the current is transmitted. After a time, however, the contractions of the inferior muscles no longer take place, save at the commencement of the action of the direct current and at the cessation of the inverse. This is extremely well shown by preparing a frog after the manner of Galvani; the two legs remaining connected with the spinal cord only by the crural nerves, and the bones of the pelvis with the lumbar vertebræ being removed. If the two legs be plunged into separate glasses of water, and the two conducting wires of the galvanic pile be plunged into these respectively, a current will pass from one glass to the other through the prepared frog, being transmitted *up* one leg, along its nerve into the spinal cord, and then *down* the other leg. Thus the same current will be *inverse* in one leg, and *direct* in the other. At first both legs contract together, when the circuit is either opened or closed; but after a time that leg only will contract on closing the circuit, in which the current is direct; whilst that leg only will contract on the interruption of the circuit, in which it is inverse. Thus the frog prepared in this manner is not merely a delicate galvanoscope, indicating the existence of a very small amount of electric disturbance; but it also serves as a galvanometer, showing the direction of the current which circulates through its nerves.

It is impossible to give a perfect demonstration of the action of the electric current upon the muscular substance; since the latter cannot be completely isolated from the nerves. But if a current of electricity be passed through a muscle, immediate contraction takes place on the closure of the circuit, whatever be the direction of the current. This contraction, however, only lasts for an instant, and does not return so long as the circuit remains closed; it usually occurs again, though less forcibly, at the interruption of the circuit; but this is not always the case, especially if the current have continued for some time, the interruption of the circuit producing no change in the state of the muscle. In no case does the direction of the current make any difference in its action on muscle; those contractions being the most powerful, and manifesting themselves for the longest period, which take place when the circuit is closed; whilst those are the weakest, and the first to disappear, which occur when it is broken. This marked departure from the laws of electrical action upon the nervous system, makes it probable that the electricity does not thus operate upon the muscle through the nerves which may remain in it, but directly upon the muscular substance itself.

It is easy to show that the diminution of the excitability of the nerve, by the continued transmission of an electric current through it, does not affect so much the entire nerve, as the part of the trunk interposed between the two conducting wires of the galvanic apparatus. For if the current be thus transmitted through a certain part of a nerve, until muscular contractions can no longer be excited by its means, the transmission of the current through a portion of the trunk at a greater distance from the brain will again call the muscles into contraction; and after this portion of the nerve has lost its excitability, the same effect may be again produced by transmitting the current through a portion of the trunk still nearer the periphery. In the same manner, when the signs of pain have ceased to manifest themselves, they may be again excited by causing the current to traverse portions of the nerve nearer and yet nearer to its central extremity, in proportion as the excitability of the trunk is weakened.

Professor Matteucci did not find any difference in the degree or duration of the excitability of the nerves after death, whether the animals had been killed by hydrogen, azote, carbonic acid, or chlorine; but he found that in animals killed by hydrocyanic acid, or by the reiterated discharges of a large battery through the spinal cord, scarcely any contraction could be excited through the nerves, although the muscles still contracted vigorously when the electric current was directly applied to them. When the animals had been poisoned by sulphuretted hydrogen, no contractions could be obtained without the employment of strong galvanic combinations; and even then they were very feeble and evanescent.

From the fact that the portion of the nerve, through which the electric current passes, loses its excitability whilst the remainder retains it, we may reasonably infer that the influence which passes through the nerve to the muscle is *not* the electric current, but something excited by it; for if it were the former, the excitability of the whole nerve would be destroyed at once. Such an inference is fully confirmed by the effects of ligatures upon the nervous trunk. If the current of electricity be transmitted through the part of the trunk round which the ligament is placed, one of the conducting wires being applied above, and the other below, the ordinary effects are manifested, in a degree somewhat weaker than usual. But if the current be wholly transmitted through the part of the trunk which is above the ligature, no *direct* muscular contractions are called forth, only the *reflex* motions being executed; whilst, on the other hand, if the current be passed through the part of the trunk below the ligature, no reflex movements are excited, but the muscular movements directly produced by the motor agency of that nerve are vigorously executed. From this circumstance it is evident, that the current of electricity transmitted through part of a nerve, is not the immediate cause of either the direct or the reflex movements; for, if it were, these movements would not be prevented as they are by the application of the ligature, which has been proved not to check an electric current. It seems evident that some particular condition of the nerve itself is the immediate stimulus to the muscular contraction, or to the reflex action of the spinal cord; and that this condition may be called forth most effectually for a time by the transmission of an electric current through a portion of the trunk. We shall hereafter find that other evidence points to the same conclusion,—the non-identity of the nervous and electric agencies.

Professor Matteucci next points out a very remarkable difference in the influence of the electric current upon the excitability of nerves, according as the course of the current be direct or inverse. We have seen that the direct current, if transmitted continuously, completely exhausts after a time the excitability of the nerve through which it passes, a period of twenty or thirty minutes being usually sufficient to effect this; but if an inverse current be made to traverse a portion of a nerve without intermission, even for three or four hours, there generally occurs, at the interruption of the circuit, a violent muscular contraction, which lasts for several seconds, and might almost be called tetanic. It ceases almost immediately upon the closure of the circuit; a new contraction at first taking place, after which the member returns to its natural condition. From this phenomenon it would appear as if the inverse current actually increases the excitability of the nerve; and such would seem to be the necessary inference from the results of other experiments of Professor Matteucci's. For he has found that if the inverse current be continued only for a fraction of a second, the contraction which takes place on breaking the circuit is decidedly less forcible than that which occurs after the current has been transmitted for one, ten, or twenty seconds, or for any more lengthened period. These effects, however, can only be demonstrated on a living animal; we cannot expect the excitability of a nerve to be increased after death. Whatever influence has been exerted upon a nerve by an electric current, a state of repose tends to restore it to its natural condition of excitability; increasing it, if it have been diminished by the direct current; or carrying off the surplus, if it have been increased by the inverse. The greater the change in the degree of excitability, the longer is the period of repose required to restore the nerve to its usual state. But when the excitability of a nerve has been suspended by the direct current, it is speedily renewed by the transmission of the inverse.

With regard to the influence of the electric current on different parts of the nervous system, we do not find anything new of importance; but it may be hoped that Professor Matteucci, having now completed his investigations in several other departments of electro-physiological inquiry will devote himself to this, with all the aid he can obtain from a previous acquaintance with the anatomical and physiological relations of the subjects of his experiments.

The difference between the effects of the continued current, and that of a frequently-interrupted current, are very remarkable; the excitability of the nerves being exhausted by the latter, much more speedily than by the former. This was first pointed out by Marianini; and has been still further substantiated by Masson, who contrived, by means of a spring pressing against a toothed wheel in revolution, to complete and interrupt the circuit many times in a second. The same result may now be obtained much more readily, however, by the electro-magnetic machine, the peculiarity of whose action consists in this continual closure and interruption of the circuit. If one of the conductors of a galvanic pile consisting of no more than ten pairs of plates, be introduced into the mouth of a rabbit, and the other communicate with the muscles of the back, and an interrupted current be then transmitted by the wheel of Masson, the animal dies within a few seconds, apparently by the exhaustion of the nervous excitability of the spinal cord. Some curious results have lately been obtained by the application of the interrupted current to the trunks of nerves, by Professor Volkmann;* of these we have already given an account elsewhere (see Vol. XXII, p. 279); but we may here notice one or two of the most interesting of his general results, which we should be glad to see confirmed by other experimenters. He distinguishes three forms of muscular contraction as produced by the rotating magnet. The first, which he terms the *continued*, is a state of tonic contraction which lasts so long as the stimulus is applied, but ceases with its withdrawal. The second continues not merely during the application of the stimulus, but even after its cessation; so that it may be termed the *persistent* contraction. In the third form there is an *alternation* of contraction and relaxation; though the stimulus remains constantly applied as before. According to Professor Volkmann, there is a relation between these forms of muscular contraction, and the mode in which the stimulus is applied. The continued contraction is produced by the passage of the current through the motor nerve itself, in the direction of the muscles; and relaxation supervenes as soon as the current ceases. The persistent contraction results from the application of the electric current to the nervous centre from which the trunk originates; and the continuance of the contraction after the cessation of the current would seem due to peculiar changes thus excited in the central organ. On the other hand, the alternation of contractions and relaxations presents itself, when the stimulus is applied to a nerve which produces muscular movement not by direct but by reflex action. If these positions be correct, we shall be supplied with a most valuable means of determining the true centres of action of many nerves, especially those belonging to the sympathetic system; but at present the generalizations of Professor Volkmann require confirmation; and we should much like to have this confirmation afforded by an experimenter on whom so much reliance may be placed as upon Professor Matteucci.

In concluding this part of his subject, Professor Matteucci makes a few observations upon the therapeutic employment of electricity. We could wish that they had been more extended; but they will suffice to show the principles which should, in his opinion, guide the selection of the particular method of applying this agent. It can scarcely be doubted that many cases of paralysis depend upon an exhaustion of the excitability of the nerves, analogous to that which is produced by the continued action of the direct current. In such cases, the action of an inverse current would seem the appropriate remedy. In favour of the employment of the current as a means of cure, he states that its passage often produces contractions in paralysed limbs; and he mentions that if the two sciatic nerves of a living frog be cut, and one of the limbs be left at rest for ten, fifteen, or twenty days, whilst the other is submitted two or three times a day to the action of the electric current, the latter will continue to exhibit con-

* Müller's Archiv, Heft v, 1845.

tractions when the former has altogether lost the power of doing so. Our readers will recollect that Dr. J. Reid tried a similar experiment, and with similar results, some years since; his object being to ascertain if the occasional exercise of a muscle paralysed by division of its nerves would maintain its nutrition and its contractility; which it was found to effect. In applying electricity therapeutically, Professor Matteucci strongly urges the importance of commencing with a very feeble current; having seen a paralytic person seized with violent convulsions of a tetanic character, on the action of a current furnished by only a single pair of plates. The action of the current should be continued for a short time only, particularly if it be energetic. If the interrupted current be employed instead of the continuous, its passage should be checked after every twenty or thirty discharges, and an interval of repose should be allowed to the patients. The application of the interrupted current would seem, in practice as in theory, to be attended with results more decidedly beneficial than those of the continued current. It is the former which is produced by the magneto-electric machine, which is, on many accounts, the most convenient means of applying electricity therapeutically.* Although, as we have seen, Professor Matteucci is sufficiently sceptical regarding many others of the alleged effects of electricity, yet he considers that the accounts of cases of paralysis, cured by the electric treatment, are already sufficiently numerous and authentic, to warrant the employment of it in cases of paralysis, with that perseverance which is necessary to warrant the hope of beneficial results.

One of the maladies in which he has proposed to employ it, is of a character that might have been supposed most completely to contra-indicate its use; namely, Tetanus. Yet theory and practice both seem to countenance its employment. The theoretical ground is simply this;—that whilst the interrupted current produces a succession of convulsive actions, the direct continued current, when sufficiently prolonged, occasions a diminution or suspension of the excitability of the nerve, and might consequently be expected to suspend tetanic convulsions, when passed through the part of the nervous system by whose undue excitability they are produced. This idea was brought to the test by Professor Matteucci upon frogs thrown into tetanic convulsions by narcotics or by hydrocyanic acid; and he states that the tetanus soon ceased under the influence of the prolonged passage of the direct current. Nevertheless the frogs died; but this was from the other effects of the narcotics employed. We should recommend the repetition of the experiment with strychnine, which will produce tetanus uncomplicated with any other change. The results obtained by the application of the electric current in a case of tetanus in the human subject, as published by Professor Matteucci in the 'Bibliothèque Universelle' (May, 1838), appear sufficiently encouraging to induce the repetition of the trial; although a favorable termination did not ensue in that instance. During the transmission of the current, the patient no longer suffered violent convulsive attacks, he became able to open and

* We would particularly direct attention to a form of this apparatus lately constructed by Mr. Hearder, a most ingenious *blind* mechanician of Plymouth. Its great advantage consists in the facility with which the nicest gradations of power may be attained; the simple movement of an index along a scale enabling the operator to increase the force of the current, by as many as thirty distinct steps, from a stream which is scarcely perceptible, to a succession of rapid shocks, of strength sufficient for any therapeutic purpose.

close his mouth, and the circulation and transpiration seemed re-established. Unfortunately, this amendment was not of long duration; for the disorder was occasioned and kept up by the presence of foreign bodies introduced into the muscles of the arm. The issue might be more favorable, where the cause of the tetanus is different; at any rate, we may advantageously have recourse to the application of electricity, for the purpose of diminishing the sufferings of the patient in this terrible malady.

It has been proposed to employ the electric current for the purpose of dissolving vesical calculi and cataract. But in neither of these cases would there appear to be any probability of success; since, even if the disintegration of the substances could be effected, the products would be perfectly insoluble and could not be removed. A cataract would be created by the coagulation of the albumen of the lens, occasioned by the passage of an electric current through its substance; but it could not be removed, since no electric action will cause the re-solution of the coagulated albumen. It has been recently proposed by Dr. Petrequin, of Lyons, to apply galvanism by means of acupuncture needles, for the cure of certain forms of aneurism; his idea being, that the electric current would occasion the coagulation of the albumen of the blood-serum, and would thus partly fill the aneurismal sac. We are not aware that this plan has been ever put in practice.

In the fourteenth and fifteenth lectures, we have an exposition of those researches of Professor Matteucci, which have been made with the view of determining the Nature and Laws of the Nervous Force. He very properly remarks that, although such a subject may appear misplaced in a treatise on the "Physical Phenomena of Life," yet there is just ground for ranking it in the same category with those other agents,—light, heat, and electricity,—which have so close a relation to each other, although it does not appear identical with any one of them. We cannot but regard the question of the identity or non-identity of the nervous force and electricity, as settled, for the present at least, by the researches of Professor Matteucci. In addition to the positive results to which we have already alluded, he has obtained many, which though of a negative character are yet very important, from experiments devised to test, in every possible way, the presence of an electric current in nerves which were exciting muscles to vigorous contraction. Although these experiments were made upon the largest nervous trunks of living animals of considerable size, and with the most delicate galvanoscopes, and altogether under circumstances most favorable to the detection of an electric current if it had any real existence, yet not the least indication of any such could be obtained. The phenomena of *induced contraction*, which were discovered by Professor Matteucci some years since, equally indicate that nervous force and electricity are two distinct agencies; at the same time that they prove the very close analogy which exists between them. Of these phenomena, the following is a brief account. If we lay the nerve of a galvanoscopic frog, (see p. 396), upon the exposed muscles of one or both thighs of a frog prepared in the ordinary manner, and cause the latter to contract by an irritation of any kind applied to the nerves or to the spinal cord, the muscles of the leg of the galvanoscopic frog are also thrown into contraction. The experiment is rendered still more striking, by arranging three or four galvanoscopic frogs in such a manner that the nerve of the second shall lie upon the muscle of the first, the nerve of the third upon the muscle of the second, and that of the fourth upon the muscle of the third. Upon irritating the nerve of the first, and throwing its muscles into contraction, the muscles of the second, third, and fourth will also be thrown into contraction, each having its action *induced* by that which precedes, and *inducing* the action in that which follows ; just as in a succession of bodies, the first of which is subjected to a disturbance of its electric equilibrium. If the nerve of the galvanoscopic frog be laid, not upon the muscle but upon the nerve of that which precedes it, no contraction is induced. And if the irritation of the nerve does not produce muscular contraction in the first animal (it is not essential that *frogs* should be employed), no action is induced in the succeeding. Now as the induced contraction takes place as well when the nerves of the first animal are excited by any other stimulus, as by electricity, it is obvious that the induction in the second cannot be explained by the passage of electricity from the first, unless that electricity be generated either in the action of the nerve or in that of the muscle. The experiments already cited prove that the action of the nerve cannot be concerned ; and by a long series of experiments, in which particular care was necessary to avoid the sources of fallacy occasioned by the muscular current, Professor Matteucci has satisfied himself that no electricity is generated by muscular contraction. Moreover he found that by interposing a thin layer of Venice turpentine between the thighs of the inducing frog and the nerve of the galvanoscopic frog laid upon them, he could prevent the passage of an electric current, without interfering with the action induced from the muscle to the nerve.

After submitting the question to every test that he could devise, he has been led to consider the phenomenon of contraction induced in muscles by the simple contact of their nerve with another muscle in the act of contracting, as a phenomenon altogether *sui generis ;* and as indicating that the action of the nervous power on a muscle produces a peculiar condition in the latter, which is capable in its turn of inducing a state of nervous excitation in the trunk of a nerve brought into simple contact with it. Thus we should be led to refer the nervous power to the same category with electricity and other polar forces, by which similar phenomena of induction are produced ; and there is this further analogy, that one of these forces may induce another. For the results of the previous experiments would lead us to the conclusion, that when muscular contractions, sensations, and other actions of the nervous system are performed in respondence to the electric stimulus, the electricity does not act directly on the muscle or on the sensorium, but excites or induces the nervous force, which is the immediate cause of the phenomena ; just as heat induces an electric disturbance in the tourmaline or in certain metallic combinations, or as light induces magnetism. The nervous force appears to have a further analogy to those with which it has been here classed, in being originated by chemical changes ; the combination of the oxygen of the arterial blood with the carbon and hydrogen of the nervous matter constituting a principal part of these changes. And when a comparison is made between the mechanical power of the human nervo-muscular system and that of any machine that man has devised, we find the former to be so much greater, in proportion to the amount of chemical change which has taken place, as to confirm the belief that the nervous force has a character altogether peculiar to itself, its mechanical operations being executed solely through living muscular tissue, but being exerted through this instrument far more powerfully than those of any other force developed by an equal amount of chemical change.

The remaining five lectures are occupied with the phenomena of muscular contraction and with the mechanism of its application in the animal body; the circulation of the blood; the action of the vocal apparatus; and the physical phenomena of hearing and sight. All these subjects are discussed, with more or less fulness, in our ordinary physiological works; and as Professor Matteucci has evidently derived the greater part of his information from these sources, especially from the laborious and detailed investigations of Müller, it is unnecessary for us to follow him through his exposition of these topics; more especially as our analysis has already reached its due limits. We shall only say that, although these lectures contain no novelty, they embrace a very clear and complete account of the several subjects embraced in them.

On the whole, then, we commend this little work to the careful study of our younger readers, with the conviction that they cannot fail to profit by it in more ways than one. It will give them a well-digested summary of the greater number of the physical actions going on in the living body; and if this summary is less complete on some points than could be desired, it is peculiarly full upon others; and whilst the former topics are for the most part of a kind on which ample information may be obtained elsewhere, the latter are such as will be almost entirely new to them, and are not readily accessible in any other form. But a still greater advantage will be derived from its perusal, if it be studied as an example of the proper method of scientific investigation, and if the success which has been attained by its philosophic author become an inducement to others to follow in the same path of inquiry with a sagacity and perseverance at all approaching his. Nor should such an example be lost upon any who are desirous of elevating the character of physiological and medical science, and whose inclinations and opportunities lead them rather to the investigation of the purely vital phenomena of living beings, than to those of a mixed or of a purely physical character. It is only by keeping constantly in view, as we have frequently urged, the general principles of scientific investigation, and by exercising yet greater caution in the analysis and comparison of results than is elsewhere necessary, in accordance with the greater complexity of the conditions under which vital phenomena occur, and the numerous sources of fallacy to which our inferences are liable, that such researches can acquire any real value; and it is consequently the more desirable that a thorough acquaintance should be first gained with works like the one now before us, which gives as good a practical lesson as could be desired on the art of observing, experimenting on, and generalizing from, the phenomena presented by living animals.

